# S100B Secretion in Astrocytes, Unlike C6 Glioma Cells, Is Downregulated by Lactate

**DOI:** 10.3390/metabo14010007

**Published:** 2023-12-21

**Authors:** Nicholas Guerini Selistre, Leticia Rodrigues, Barbara Carolina Federhen, Vitor Gayger-Dias, Jéssica Taday, Krista Mineia Wartchow, Carlos-Alberto Gonçalves

**Affiliations:** 1 Biochemistry Post-Graduate Program, Federal University of Rio Grande do Sul (UFRGS), Porto Alegre 90010-150, Brazil; nicholas.gueriniselistre@gmail.com (N.G.S.); letigues@gmail.com (L.R.); barbarafederhen@gmail.com (B.C.F.); vitor.dias@ufrgs.br (V.G.-D.); jhtaday@gmail.com (J.T.); casg@ufrgs.br (C.-A.G.); 2 Brain Health Imaging Institute, Department of Radiology, Weill Cornell Medicine, New York, NY 10044, USA

**Keywords:** astrocytes, hippocampal slices, fluorocitrate, glucose metabolism, C6 cells, lactate

## Abstract

S100B is a calcium-binding protein produced and secreted by astrocytes in response to various extracellular stimuli. C6 glioma cells are a lineage commonly employed for astroglial studies due to the expression of astrocyte specific markers and behavior. However, in high-glucose medium, C6 S100B secretion increases, in contrast to the trend in primary astrocyte cultures. Additionally, S100B secretion decreases due to fluorocitrate (FC), a Krebs cycle inhibitor, highlighting a connection between S100B and metabolism. Herein, we investigate the impact of FC on S100B secretion in primary astrocyte cultures, acute hippocampal slices and C6 glioma cells, as well as lactate mediation. Our results demonstrated that C6 responded similarly to astrocytes in various parameters, despite the decrease in S100B secretion, which was inversely observed in astrocytes and slices. Furthermore, FC inversely altered extracellular lactate in both models, suggesting a role for lactate in S100B secretion. This was reinforced by a decrease in S100B secretion in hippocampal slices treated with lactate and its agonist, but not in C6 cells, despite HCAR1 expression. Our findings indicate that extracellular lactate mediates the decrease in S100B secretion in astrocytes exposed to FC. They also emphasize the differences in C6 glioma cells regarding energetic metabolism. The proposed mechanism via HCAR1 provides further compelling evidence of the relationship between S100B and glucose metabolism.

## 1. Introduction

C6 glioma cell lines, depending on the cell passage, are commonly used as astroglial cells due to the expression and activity of specific astrocytic parameters [1]. This allows the use of these cells in a variety of experimental and preclinical, biochemical and pharmacological assays [2,3,4].

One of these astrocytic parameters is the secretion of S100 calcium-binding B protein (S100B). This protein has a homodimeric organization and weighs 21 kDa. In the intracellular environment, it has several substrates, such as glial fibrillary acid protein (GFAP), AHNAK (a scaffold protein that anchors L-type calcium channels), enzymes such as phosphoglucomutase and proteins involved in gene transcription such as p53 [5,6]. For both astrocytes and C6 glioma cells, S100B is constitutively secreted and can, in situations of injury, be more actively released, acting as an alarmin on RAGE, the receptor for both glycation end-products and S100B itself [7].

Indeed, astrocytes and C6 glioma cells respond similarly to many harmful stimuli, such as LPS, with respect to TNFα and even S100B secretion [1,8,9,10]. However, S100B secretion in astrocytes and C6 cells behaves oppositely when exposed to a medium with a high glucose concentration. Astrocytes reduce S100B secretion, while C6 cells increase it [11]. C6 glioma cells, being more proliferative, have a different oxidative metabolism for glucose than astrocytes. Perhaps this difference is related to the different S100B secretion profiles. It is well established that C6 glioma cells take up more glucose and release proportionally less lactate [1,12,13].

Primary astrocytes treated with fluorocitrate (FC) increase glucose uptake and decrease S100B secretion [14]. FC is an aconitase inhibitor (blocking the Krebs cycle), which is taken up into cells by the lactate transporter MCT1. This, in the CNS, makes astrocytes almost specific targets for FC [15,16]. FC increases glucose uptake due to an increase in the Warburg-type effect. Furthermore, more recently, it has been observed that lactate generated in the glycolytic pathway can also modulate cellular signaling through specific receptors (e.g., HCAR1) [17,18] Decreased S100B secretion is also observed in astrocytes in hippocampal slices treated with FC [14,19]. Thus, we hypothesized that C6 cells exposed to FC, due to differences in glucose oxidative metabolism compared to astrocytes, display opposite behavior in relation to S100B secretion compared to what they displayed when exposed to a medium with high glucose. In fact, the S100B secretion profiles were opposite for C6 glioma cells and astrocytes in primary cultures and hippocampal slices. Herein, we detail this difference and point to the possible mechanism involved.

## 2. Materials and Methods

### 2.1. Chemicals

Dulbecco’s modified Eagle medium (DMEM), fetal calf serum (FCS), Hank’s balanced salt solution (HBSS) and other materials used for cell cultures were purchased from Gibco BRL (Carlsbad, CA, USA). 3,5-Dihydroxybenzoic acid (DHBA), Methylthiazolyldiphenyl-tetrazolium bromide (MTT), DL-Fluorocitric acid barium salt, cytochalasin B, GSH standard stock solution, 4-(2-hydroxyethyl) piperazine-1-ethanesulfonic acid (HEPES), o-phthaldialdehyde, γ-glutamylhydroxamate, o-Phenylenediamine, S100B protein, anti-S100B antibody (SH-B1) and anti-GPR81-S296 antibody were purchased from Sigma Aldrich (St Louis, MO, USA). Peroxidase secondary antibody was purchased from GE (Little Chalfont, United Kingdom). Anti-β-actin conjugated with HRP was purchased from Protein Tech (Chicago, IL, USA). Polyclonal anti-S100B (clone SH-B) was purchased from Dako (São Paulo, Brazil). Deoxy-[_3-3_H] glucose (20 Ci/mmol) and l-[_3,4-3_H] glutamate (0.33 μCi/mL) were purchased from PerkinElmer (Boston, MA, USA). Non-described reagents were purchased from local commercial suppliers (Sulquímica or Labsul, Porto Alegre, Brazil).

### 2.2. Animals

For the preparation of hippocampal slices and primary astrocyte culture, we used male 30-day-old Wistar rats and newborn, 1–2-day-old Wistar rats obtained from our breeding colony (Department of Biochemistry, UFRGS, Porto Alegre, Brazil). The animals were maintained under controlled light and environmental conditions (12 h light/12 h dark cycle at a constant temperature of 22 ± 1 °C) with free access to commercial food and water. The procedures were carried out in accordance with the NIH Guide for the Care and Use of Laboratory Animals and approved by the local authorities.

### 2.3. Acute Hippocampal Slices

Rats were decapitated, and their hippocampi were quickly dissected out. Transverse sections (0.3 mm) of tissue were rapidly obtained using a McIlwain tissue chopper. One slice was placed into each well of a 24-well culture plate. Slices were incubated in oxygenated physiological medium containing 120 mM NaCl, 2.0 mM KCl, 1.0 mM CaCl_2_, 1.0 mM MgSO_4_, 25.0 mM Hepes, 1.0 mM KH_2_PO_4_ and 10.0 mM glucose, with a pH of 7.4, at room temperature. The medium was replaced every 15 min with fresh medium. Following a 120 min equilibration period, slices were incubated in medium in the presence/absence of treatment conditions for 1 h at 30 °C [20].

### 2.4. Primary Astrocytes Culture

The primary astrocyte culture protocol was performed as previously described [21]. In brief, cerebral cortices of newborn rats were removed and mechanically dissociated in Dulbecco’s Phosphate Buffered Saline (DPBS) without Ca^2+^ and Mg^2+^ at pH 7.4 (containing 137 mM NaCl; 5.36 mM KCl; 0.27 mM Na2HPO_4_; 1.1 mM KH2PO_4_ and 6.1 mM glucose). The cortices were cleaned of meninges and mechanically dissociated via sequential passage through a Pasteur pipette. After centrifugation at 300× *g* for 5 min, the pellet was suspended in DMEM low glucose (pH 7.6) supplemented with 8.39 mM HEPES, 23.8 mM NaHCO_3_, 0.1% amphotericin B, 0.032% gentamicin and 10% fetal calf serum (FCS). Cells were plated into 24-well plates (300.000 cells/well) pre-treated with poly-L-lysine. Cultures were maintained in DMEM containing 10% FCS in 5% CO_2_/95% air at 37 °C. The medium was changed every 3–4 days. Cells were allowed to grow to confluence and used after 21 days in vitro. The purity of astrocyte primary cell culture was more than 95%, as assessed via immunocytochemistry (GFAP/S100B double staining). We were unable to label neurons or microglia using anti-NeuN or anti-Iba-1, respectively.

### 2.5. C6 Glioma Cell Culture

The C6 glioma cell line was obtained from the American Type Culture Collection (Rockville, MA, USA) and cultured according to a previously described procedure [9,10,22,23]. To allow us to use C6 glioma cells as an astroglial model, they reached the late passage stage (after at least 100 passages) and were then seeded in flasks and cultured in DMEM low glucose (pH 7.4) containing 5% FCS, 0.1% amphotericin B and 0.032% gentamicin. Cell cultures were maintained at a temperature of 37 °C in an atmosphere of 5% CO_2_ and 95% air. At confluence, cells were detached from the culture flasks with 0.05% trypsin/EDTA and seeded (5 × 10^3^ cells/cm^2^) in 24-well plates. These plates were maintained under the same flask conditions for 3 days or until reaching confluence to start treatments.

### 2.6. Treatments

The glioma C6 cultures, primary astrocyte cultures and acute hippocampal slices were treated with different concentrations of FC (1, 10 and 100 μM) [14,24,25,26], 2 mM L-Lactate [27] and 2 mM DHBA [28]. C6 glioma and astrocytes cultures were treated by removing the medium and incubating the cells with compounds in DMEM without FBS for 1 h at 37 °C in an atmosphere containing 5% CO_2_/95% air. Acute hippocampal slices were treated by removing the HBSS, replacing it with the same new medium and incubating them with compounds for 1 h at 30 °C at the end of the equilibration period.

### 2.7. Cell Viability and Integrity

#### 2.7.1. MTT Reduction Assay

After the FC treatment was completed, MTT solution was added (50 μg/mL), and the cell culture was incubated for 30 min at 37 °C. Afterward, the medium was removed, and the MTT crystals produced in viable mitochondria were dissolved in dimethyl sulfoxide (DMSO). Absorbance values were measured at 560 and 650 nm. The results are expressed as percentages of the control value [22].

#### 2.7.2. Lactate Dehydrogenase Assay

The presence of lactate dehydrogenase (LDH) in the extracellular medium was determined using a commercial colorimetric assay sourced from BIOCLIN™ (São Paulo, Brazil) using 100 µL of the sample. The results are expressed as percentages of the control value [22].

### 2.8. Glucose Uptake Assay

Glucose uptake was performed as previously described [10,29], albeit with some modifications. After treatment, the medium was removed, and C6 glioma cells were incubated at 35 °C in Hank’s balanced salt solution (HBSS) with the addition of 0.1 μCi/well D-[2,3-^3^H] deoxy-glucose for 15 min. The incubation was stopped by removing the medium and rinsing the plate wells with ice-cold HBSS. The cells were then lysed in a 0.5 M NaOH solution. Non-specific glucose uptake was performed using the glucose transporter inhibitor cytochalasin B (25 μM) and utilized to calculate final uptake by subtracting from the total uptake. Radioactivity was measured using a scintillation counter, and the results are expressed as percentages of the control value.

### 2.9. Extracellular Lactate Assay

The presence of L-lactate in the extracellular medium was determined via a commercial colorimetric assay from BIOCLIN™ (Brazil) using 150 µL of the sample. The results are expressed as percentages of the control value.

### 2.10. Glutamate Uptake

The glutamate uptake assay was performed as previously described by using H3-labeled glutamate [30]. In brief, C6 glioma cell culture was incubated at 35 °C in Hank’s balanced salt solution (HBSS). Posteriorly, 0.1 mM L-glutamate and 0.33 μCi/mL l-[_3,4-3_H] glutamate were added to the medium to initiate the assay. After 10 min, the incubation was stopped by removing the medium and rinsing the plate wells with ice-cold HBSS, and the cells in the plate wells were then lysed in a 0.5 M NaOH solution. We then performed sodium-independent uptake (non-specific) using ice-cold HBSS without sodium (used N-methyl-d-glucamine instead of sodium chloride). Measurements were obtained using a scintillation counter and by subtracting the sodium-dependent uptake from the total uptake. The results are expressed as percentages of the control value.

### 2.11. Glutamine Synthetase Activity

GS activity was determined as previously described [31]. In brief, cells were lysed and then added to homogenate a reaction mixture containing 10 mM MgCl_2_, 50 mM L-glutamate, 100 mM imidazole–HCl buffer, 10 mM 2-mercaptoethanol and 50 mM hydroxylamine–HCl. To start the reaction, we added 10 mM ATP, which was continued for 15 min at 37 °C. To stop the reaction, we used a solution containing 370 mM ferric chloride, 670 mM HCl and 200 mM trichloroacetic acid. After this, the homogenate was centrifuged, and absorbance values were measured in the supernatant at 530 nm. A standard/calibration curve was prepared using a fixed concentration of γ-glutamyl hydroxamate and treated with a ferric chloride reagent. The results are expressed as percentages of the control value.

### 2.12. Glutathione Levels

Intracellular GSH levels were measured as previously described [32] in a homogenate obtained by C6 glioma cell lysates suspended in a mixture solution of sodium phosphate (100 mM), KCl buffer (140 mM) and EDTA (5 mM). To precipitate proteins, we added 1.7% meta-phosphoric acid and performed centrifugation. Next, the samples (supernatant) and calibration curve (GSH solutions, 0 to 500 μM) were incubated with o-phthaldialdehyde (at a concentration of 1 mg/mL diluted in methanol) at 22 °C for 15 min. The fluorescence was measured using excitation and emission wavelengths of 350 and 420 nm, respectively. The results are expressed as percentages of the control value.

### 2.13. ELISA for S100B

S100B secretion was measured via an enzyme-linked immunosorbent assay (ELISA) based on the work published by Leite et al., 2008 [33]. In brief, 50 μL of the sample (extracellular medium) plus 50 μL of Tris buffer were incubated on a microtiter plate previously coated with anti-S100B monoclonal antibody (SH-B1, from Sigma) for 2 h. Anti-S100 polyclonal antibody (from DAKO) was incubated for 30 min, and peroxidase-conjugated anti-rabbit antibody was added for a further 30 min. The colorimetric reaction with o-phenylenediamine (OPD) was measured at 492 nm. The standard S100B curve ranged from 0.002 to 1 ng/mL. The results are expressed as percentages of the control value [33].

### 2.14. Western Blotting

Cell culture samples were homogenized in sample buffer (62.5 mM Tris–HCl, pH 6.8, 10% (*v*/*v*) glycerol, 2% (*w*/*v*) SDS, 5% (*w*/*v*) β-mercaptoethanol and 0.002% bromophenol blue) and applied equally for separation at a concentration of 20 µg of total protein on SDS PAGE on 10% (*w*/*v*) acrylamide gel and electro-transferred onto nitrocellulose membranes. Membranes were incubated in TBS-T (20 mmol/L Tris–HCl, pH 7.5, 137 mmol/L NaCl, 0.05% (*v*/*v*) Tween 20) containing 5% (*w*/*v*) bovine serum albumin (BSA) for 1 h at room temperature. Subsequently, the membranes were incubated overnight with antibody anti-GPR81 (dilution 1:1000) (Sigma Aldrich), rinsed with TBS-T and exposed to horseradish peroxidase-linked (HRP) anti-IgG antibody for 2 h at room temperature. For the detection of β-actin, we used anti-β-actin conjugated with HRP (dilution 1:30,000). Chemiluminescent bands were detected using Bio-Rad^®^ ChemiDoc MP (Bio-Rad® Hercules, CA, USA), and densitometry analyses were performed using Image Lab 6.1 software. The results were expressed as percentages of the control.

### 2.15. Immunofluorescence

The cells were cultured on circular glass coverslips without any treatment and fixed for 20 min with 4% paraformaldehyde in phosphate buffer (PBS), washed with PBS and permeabilized for 20 min in PBS containing 0.2% Triton X-100. The cells were then blocked for 1 h with PBS containing 5% bovine serum albumin and incubated overnight with anti-GPR81-S296 at a 1:500 dilution. Following overnight incubation, cells were washed in PBS/triton 0.2% (3 × 5 min) and incubated for 2 h with the respective secondary antibodies at a 1:1000 dilution—Alexa Fluor 528 (goat anti-mouse-IgG; red fluorescence). Images were captured using an Olympus BX51 phase-contrast fluorescence microscope (Olympus, Tokyo, Japan) and transferred to a computer using a digital camera and Fluoviewer 3.1 FV1000 software for analysis [24].

### 2.16. Protein Determination

Protein content was measured via Lowry’s method, albeit with some modifications, using bovine serum albumin as the standard [34].

### 2.17. Statistical Analysis

Each set of results was obtained from at least four independent experiments performed in triplicate. Student’s *t* test was performed to statistically analyze differences between two groups and one-way analyses of variance (ANOVA), and Tukey’s test was then performed to perform statistically analyses when there were three or more groups. All analyses were performed using Graphpad Prism^®^ 8 software. Values of *p* < 0.05 were considered significant. a indicates similarity to basal conditions, while b and c indicate the difference from basal conditions. * indicates differences between two groups.

## 3. Results

### 3.1. S100B Secretion Differs in C6 Glioma Cells and Astrocytes

**Figure 1:**
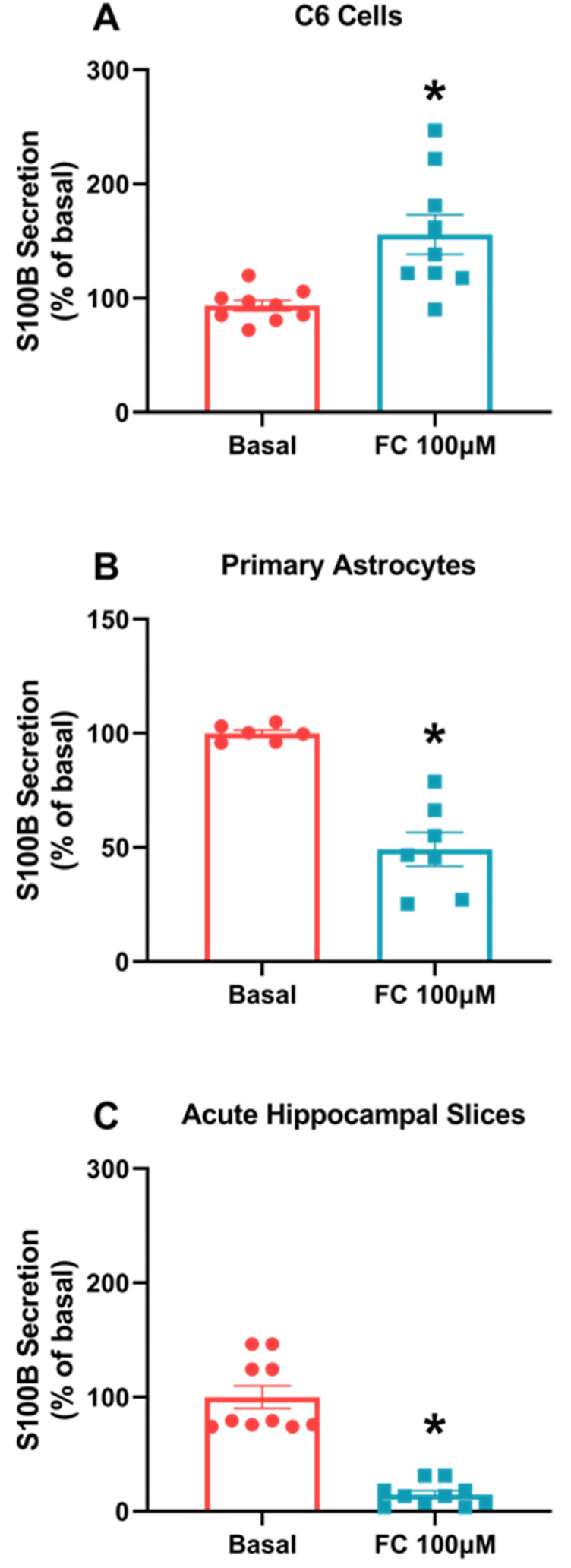
FC differently alters S100B secretion in C6 glioma cells, astrocytes and acute hippocampal slices. S100B secretion was measured via ELISA after 1 h of FC treatment in the extracellular media of C6 cells (**A**), astrocytes (**B**) and acute hippocampal slices (**C**). Values are mean ± standard error. * indicates differences from basal conditions (n = 6–10, unpaired Student’s *t* test).

S100B is a major protein in astrocytes, so its secretory profile responds in face a variety of insults. Here, we observed different responses between the two models. In C6 glioma cells, there was an increase in S100B protein secretion compared to the basal group (Figure 1A, *p* = 0.0032), while in primary astrocyte cultures’ (Figure 1B, *p* = 0.0002) acute hippocampal slices, we observed a decrease in S100B secretion compared to the basal group (Figure 1C, *p* < 0.0001).

### 3.2. FC Concentrations Do Not Alter the Viability and Integrity of C6 Glioma Cells

**Figure 2:**
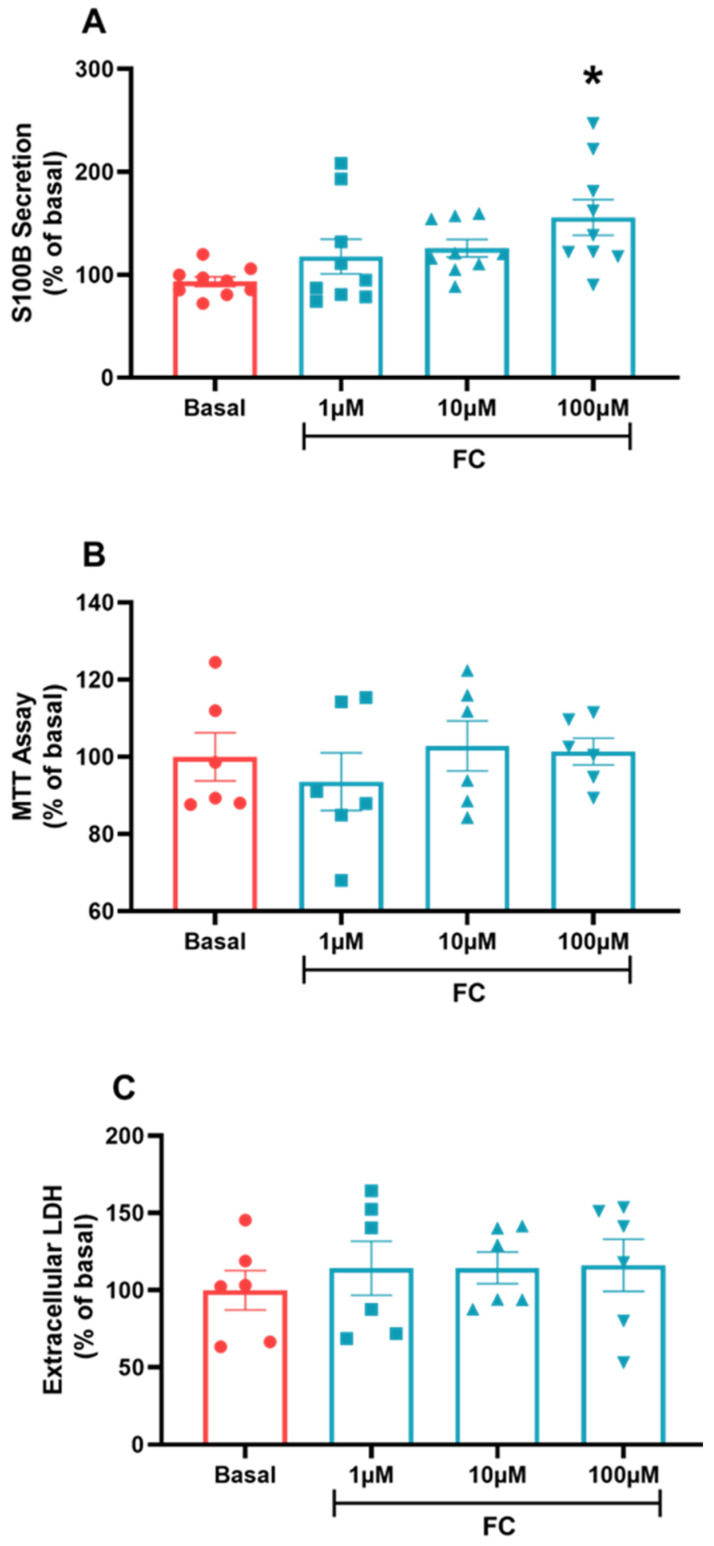
C6 glioma cells treated via the FC curve. To evaluate the secretion of S100B, (**A**) extracellular medium was collected and measured via ELISA. MTT assay (**B**) was used to evaluate cellular viability. Extracellular LDH (**C**) was measured in the extracellular medium to evaluate cellular integrity. Values are mean ± standard error. * indicates differences from basal conditions (n = 6–8, one-way ANOVA, followed by Tukey’s test).

To establish the most effective response concentration required to treat C6 glioma cells based on other in vitro models, we chose a FC concentration curve to treat C6 glioma cells in a range among 1, 10 and 100 µM (the highest concentration being the most used in other in vitro works). We then observed that only the highest concentration had a significant effect on S100B secretion, increasing this parameter compared to the basal group (Figure 2A, *p* = 0.0175, F_(3, 32)_ = 2.08). In addition, we performed a cell viability and integrity test, an MTT and an extracellular LDH assay. Our results indicated that there was no toxic effect or damage that could lead to loss of cell viability and integrity at any of the concentrations used (Figure 2B, *p* = 0.7214, F_(3, 20)_ = 0.9130; Figure 2C, *p* = 0.8504, F_(3, 20)_ = 1.227), which allows us to use 100 µM in other tests and assays.

### 3.3. Some Astrocytic Parameters in C6 Glioma Cells Change in a Similar Manner to Astrocytes in Cultures When Exposed to FC

**Figure 3:**
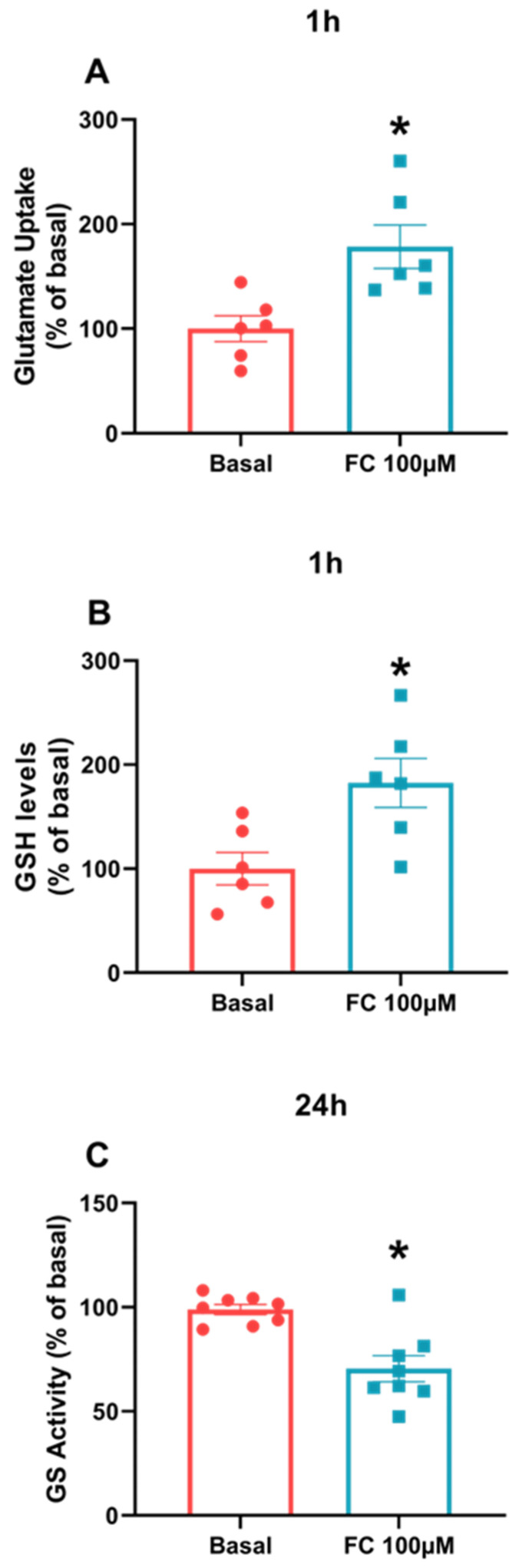
Astrocytes parameters measured in C6 glioma cells treated with FC. To evaluate glutamate uptake (**A**), GSH levels (**B**) and GS activity (**C**), cell culture was lysed in a specific buffer for each assay. Values are mean ± standard error. * indicates differences from basal conditions (n = 6–8, unpaired Student’s *t* test).

Despite the difference in S100B secretion between C6 glioma cells and acute hippocampal slices, we evaluated other astroglial parameters in C6 cells exposed to FC, such as glutamate uptake, glutamine synthetase activity and GSH content. The purpose of this approach was to determine whether this cell type behaves like astrocytes, as seen in other data published in the literature. As a result, we observed that in the three parameters, analyzed C6 glioma cells behave like astrocytes, since the results showed increases in glutamate uptake (Figure 3A, *p* = 0.0087) and GSH content (Figure 3B, *p* = 0.0155) (after 1 h of treatment) and GS activity decreases (after 24 h of treatment) (Figure 3C, *p* = 0.0008) compared to the baseline groups.

### 3.4. FC Alters Glucose Uptake but Responds Differently to Lactate Metabolism in C6 Glioma Cells and Hippocampal Slices

**Figure 4:**
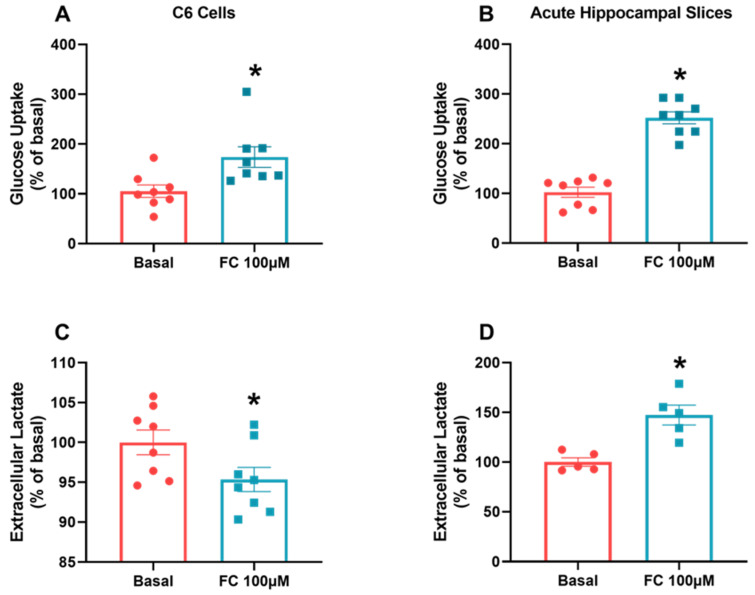
FC induces an increase in glucose metabolism but different responses in extracellular lactate in C6 glioma cells and acute hippocampal slices. To evaluate glucose uptake (**A**,**B**), in vitro models were incubated with D-[2,3-^3^H] deoxy-glucose for 15 min and then lysed. For extracellular lactate, measurements (**C**,**D**) were collected from extracellular medium after ending the treatment period. Values are mean ± standard error. * indicates differences from basal conditions (n = 5–8, unpaired Student’s *t* test).

To more closely examine the observed difference in S100B secretion related to oxidative metabolism, we evaluated glucose uptake in and lactate release into the extracellular medium in C6 glioma cells and hippocampal slices in the presence of FC. Glucose uptake enabled increases in both C6 glioma cells (Figure 4A, *p* = 0.0132) and hippocampal slices (Figure 4B, *p* < 0.0001) when exposed to FC. On the other hand, the extracellular lactate concentration decreased in C6 glioma cells when exposed to FC (Figure 4C, *p* = 0.0496) but increased in hippocampal slices (Figure 4D, *p* = 0.0024).

### 3.5. HCAR1 Could Be Involved in S100B Secretion in Astrocytes but Not in C6 Glioma Cells

**Figure 5:**
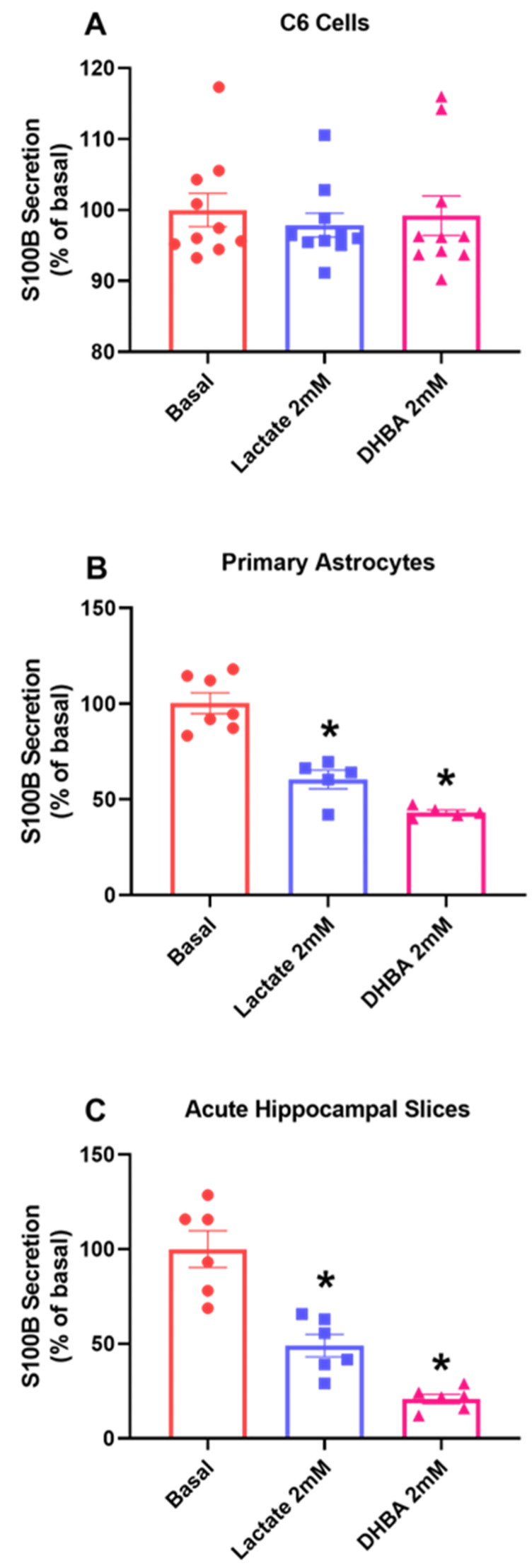
C6 glioma cells respond differently to HCAR1 stimulus compared to acute hippocampal slices. After 1 h treatment with lactate and the HCAR1 agonist receptor DHBA, extracellular medium was collected to evaluate the secretion of S100B in C6 glioma cells (**A**) primary astrocytes (**B**) and acute hippocampal slices (**C**). Values are mean ± standard error. * indicate differences from basal conditions. (n = 6–10, one-way ANOVA, followed by Tukey’s test).

At this point, it became clearer that lactate, at least in part, could mediate this difference in relation to S100B secretion. To better understand this mechanism, we treated the different cell preparations with lactate and DHBA, an HCAR1 agonist. In C6 glioma cells, basal secretion was not modified by the presence of lactate or DHBA (Figure 5A, *p* = 0.8067, F_(2, 27)_ = 0.4313). In contrast, S100B secretion in primary astrocyte culture (Figure 5B, *p* < 0.0001, F_(2, 14)_ = 2.439) and acute hippocampal slices (Figure 5C, *p* < 0.0001, F_(2, 15)_ = 7.348) was altered by lactate, reducing protein basal secretion and the lactate agonist DHBA.

### 3.6. C6 Glioma Cells Normally Expressed HCAR1

**Figure 6:**
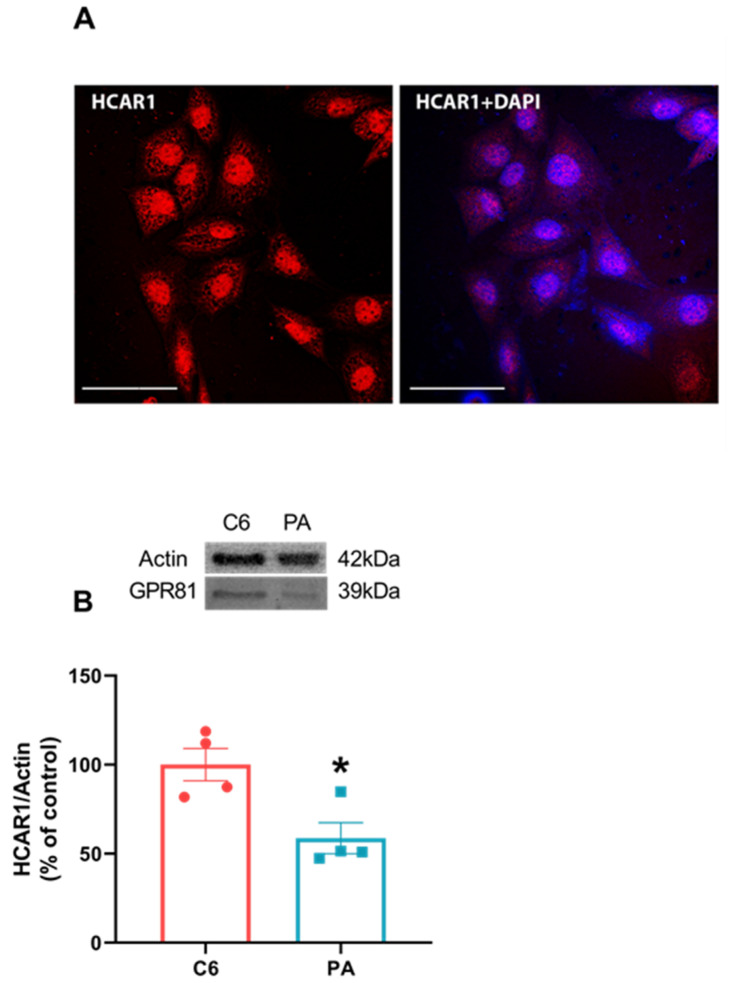
C6 glioma cells express HCAR1 more than primary astrocytes cultures. C6 glioma cells were seeded in circular glass covers slips without any treatment and fixed for visualization in confocal microscope. A representative image for the expression of HCAR1 (red) and merging with DAPI in C6 glioma cells via immunofluorescence using specific antibodies (**A**). A 60× magnitude with a scale bar of 50 µm. C6 glioma cells and primary astrocytes cultures (PA) were seeded in a 6-well plate and, at confluence, were prepared samples for Western blotting and applied equally at a concentration of 20 µg total protein for HCAR1 content (**B**). Values are mean ± standard error. * indicates difference between two groups. (n = 4, unpaired Student’s *t* test).

Considering the discrepant behavior between astrocytes and C6 glioma cells in some aspects, we investigated the distribution of HCAR1 expression and content in C6 cells. We thought that its absence might possibly explain the fact that C6 glioma cells do not respond to lactate and DHBA treatments. However, we can see via immunofluorescence that C6 glioma cells express HCAR1 (Figure 6A), and we compared this cell type with a primary astrocyte culture via Western blotting, finding that C6 glioma cells have more HCAR1 content than primary astrocyte cultures (Figure 6B, *p* = 0.0168).

## 4. Discussion

Lineages of C6 glioma cells, depending on the cellular passage, have been used as equivalent models to investigate astrocyte behavior [35]. In this study, we used FC as a gliotoxin to assess changes in specific astroglial parameters in C6 glioma cells, cultured astrocytes and astrocytes derived from acute brain slices. These in vitro preparations have been widely used for drug and compound toxicity screening. Indeed, changes in specific glial parameters such as glutamate uptake, GSH content and glutamine synthetase in C6 cells exposed to FC were equivalent to those described for astrocytes in primary culture or acute hippocampal slices (see Figure 3). This reinforces the importance of these C6 glioma cells as astrocyte equivalents for use in drug screening, coupled with the ease of cell cultures.

However, if we look at S100B secretion, C6 glioma cells exposed to FC behaved in an opposite manner to astrocytes. FC promoted increased S100B secretion in C6 glioma cells, while in reduced in astrocyte cultures and acute hippocampal slices (see Figure 1).

FC have been classically used for astrocytic inactivation, resulting in pathophysiological studies of Alzheimer’s disease, neuroinflammation, ischemia, depression and traumatic brain injury, in order to both understand cellular mechanisms and promote therapeutic strategies [36,37,38,39,40,41,42]. This compound targets the aconitase enzyme, binding strongly to it and then interrupting the Krebs cycle. However, the resulting blockage of ATP production stimulates cytosolic glycolysis (known as the Warburg effect). Cell-dependent effects/toxicity result from FC entering astrocytes and C6 cells via the glial lactate transporter 1 (MCT1), which also transports FC, restricting its action to glial lineages once neuronal cells contain the enzyme but express other types of MCTs [15,43,44].

A few years ago, we observed that astrocytes and C6 glioma cells expressed and secreted S100B in an opposite way when exposed to a medium with high glucose [11]. In this work and echoing previous results from our laboratory, a contrary behavior after FC exposure was also found in S100B secretion, reinforcing the idea of differentiated metabolization or the signaling of glucose [14,24]. Nevertheless, the secretion mechanism of S100B is not yet known [45]. Whether this mechanism is related to glucose metabolism in astrocytes is unknown, but we know that extracellular S100B somehow affects glucose metabolism [10].

Confirming observations from the literature, FC increased glucose uptake occurs, both in C6 glioma cells and acute brain slices. The increase in astrocyte glucose uptake is possibly associated with glutamate uptake and the increase in GSH synthesis [23]. Furthermore, the reduction in alpha-keto-glutarate caused by FC will later be reflected in the reduction in GS activity [15,46,47], which we observed at 24 h but not 1 h treatment. Astrocytes have high levels of GSH to protect the CNS from the consequences of intense oxidative metabolism. They produce and secrete GSH, which even serves as a substrate for neuronal GSH. Gliomas also have high levels of GSH and need to protect themselves from oxidative metabolism by neighboring cells and possible attacks by the immune system [1,48,49].

Regarding lactate exports (see Figure 4), we also observed differences between astrocytes and C6 glioma cells when exposed to FC. The lactate export profile observed in hippocampal slices is similar to those of cultured astrocytes. Astrocytes increase extracellular lactate, reflecting the increased glucose uptake. On the contrary, C6 glioma cells, even though they capture more glucose, export less lactate. These more proliferative cells seem to be unable to meet neighboring cells’ energy demands. In C6 glioma cells, it is possible that MCT1 is functionally coupled to NHE1 to acidify the surrounding environment [50].

**Figure 7:**
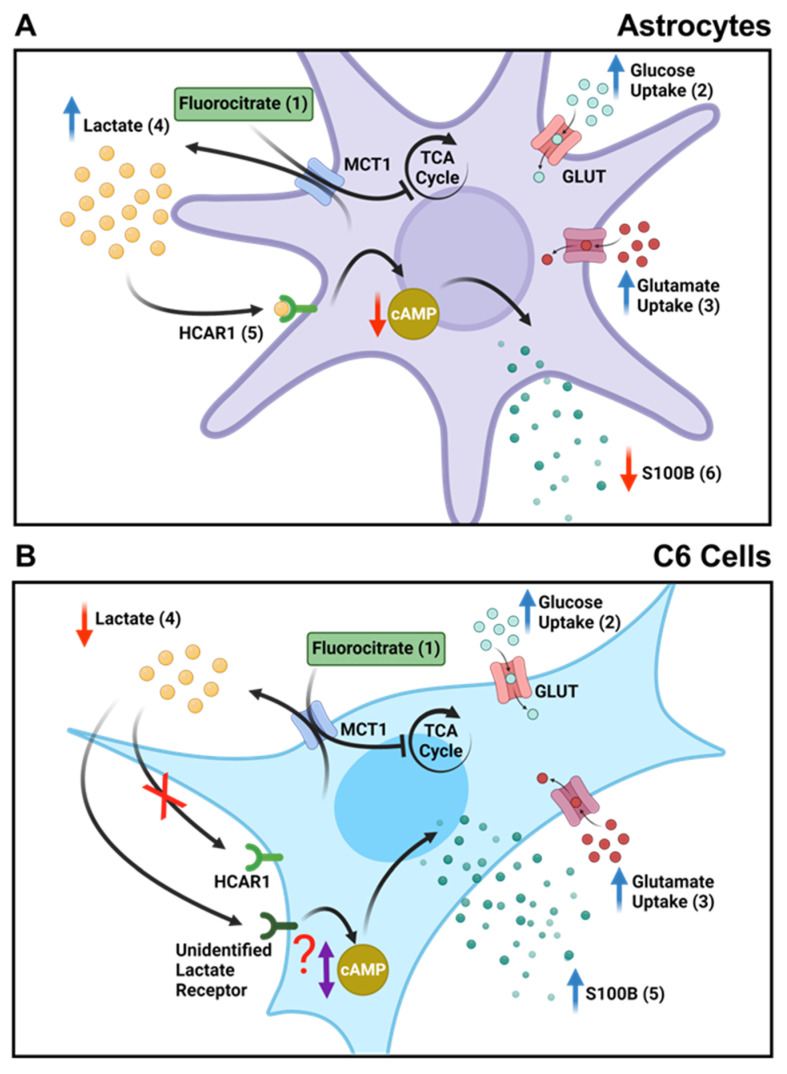
FC reduces the secretion of S100B in astrocytes and acute hippocampal slices but not in C6 glioma cells. (**A**) In astrocytes, (1) FC enters cells through MCT1 and inhibits the TCA cycle, acting directly on the enzyme aconitase. (2) This leads to an increase in glucose uptake as a compensatory action for the inhibition of energy metabolism caused by FC. (3) This also results in increased glutamate uptake through the same metabolic compensation mechanism. (4) Due to the inhibited TCA cycle, anaerobic respiration increases, leading to the higher production and extracellular release of lactate. (5) Elevated extracellular lactate levels bind to the HCAR1 receptor, which is coupled to a Gi protein, reducing cAMP levels. (6) Finally, a decrease in S100B secretion occurs due to cAMP levels changes. (**B**) In C6 glioma cells, (1) FC also enters through MCT1 and similarly inhibits the TCA cycle. (2) There is also an increase in glucose and glutamate uptake as a compensatory measure in cellular metabolism. (3) Additionally, the glutamate taken up by the cells is directed towards increasing the production of GSH as a cellular defense against the effects of FC, and there is a decrease in GS activity, possibly due to decreased ATP levels. (4) Unlike astrocytes, there is a decrease in the extracellular release of lactate in C6 glioma cells. (5) This difference is also observed in S100B secretion, where FC causes an increase in C6 glioma cells. This indicates that lactate signaling may not be important for this cell type or may occur through another receptor or mechanism that may or may not alter cAMP levels.

This difference in extracellular lactate could underlie the difference in S100B secretion between C6 and astrocytes. In fact, we know that lactate is more than an energy metabolite, as through specific receptors, it can signal and modulate cellular functions [18,51,52]. Once the lactate receptor HCAR1 (Hydroxycarboxylic acid receptor 1), formerly known as G protein-coupled receptor 81 (GPR81), is coupled to a G inhibitory protein (Gi), which is in turn coupled to adenylyl cyclase (AC), we can assume that extracellular lactate could lead to a reduction in cAMP levels (see Figure 7). We know that S100B secretion in astrocytes is positively modulated by cAMP [53,54]. In this case, lactate via HCAR1 could reduce S100B secretion. Indeed, this mechanism explains the reduction in S100B secretion caused by FC in cultured astrocytes and acute hippocampal slices. Both lactate and DHBA, an HCAR1 agonist, reduced S100B secretion (Figure 5). In support of this view, forskolin (an AC activator), in hippocampal slices, increased S100B secretion. Furthermore, this increase was antagonized by lactate and DHBA.

This mechanism of reducing S100B secretion mediated by lactate can be seen in situations of excitotoxicity [20,53,55,56,57]. High glutamate leads to a reduction in cAMP and reduced S100B secretion [53]. In injury situations, such as ischemia, high levels of glutamate contribute to an increase in lactate released by astrocytes, which, through HCAR1, attenuate cAMP levels and Ca^2+^ influx, promoting neuroprotection in astrocytes [56]. Indeed, lactate appears able to antagonize the effect of glutamate on S100B secretion in brain slices [58].

Even so, this mechanism does not seem to exist or be functional in C6 glioma cells. Increased levels of extracellular lactate or DHBA did not modify S100B secretion. Based on that finding, our next step was to investigate whether C6 cells express HCAR1 in comparison to astrocytes. Its absence could be involved in the S100B secretion modulation and explain this variation. Surprisingly, C6 glioma cells present a higher expression of the HCAR1 receptor than primary astrocyte culture (Figure 6). These data reinforce the difference in lactate-mediated signaling between astrocytes and C6 glioma cells, and this difference is now reported for the first time in the literature. However, it does not explain the increase in S100B secretion in C6 glioma cells in the presence of FC (see Figure 7). Furthermore, the immunocytochemical image of HCAR1 points to the presence in the plasma and perinuclear membrane of this receptor in C6 glioma cells. This does not clarify the activity or functionality of this receptor.

This doubt allows at least two predictions. One is that lactate signaling via HCAR1 for S100B secretion is less important in C6 glioma cells. Another possibility is that the transduction mechanism related to lactate is different in those cells. It has already been suggested that lactate could increase cAMP and/or have its action measured by another receptor, in addition to HCAR1 [48,56]. These possibilities remain open for C6 glioma cells. However, S100B secretion is complex, and there are many secretagogues in addition to lactate. The participation of the HCAR1 receptor will require further experiments with knockout or silenced cells for this receptor.

## 5. Conclusions

This is the first time that extracellular lactate, via HCAR1, has been shown to mediate the reduction in S100B secretion in astrocytes exposed to FC. This reinforces the difference between astrocytes and C6 glioma cells regarding glucose metabolism/signaling, as the proposed mechanism is different or secondary to that of C6 glioma cells. The proposed mechanism of modulation of S100B secretion via HCAR1 broadens our understanding of the relationship between protein and glucose metabolism, as well as our view of the extracellular levels of S100B in brain energy disorders.

## Data Availability

The data used to support the findings of this study are available from the corresponding author upon request. Data it not publicly available due to privacy.
